# Itraconazole Reversing Acquired Resistance to Osimertinib in NSCLC by Inhibiting the SHH/DUSP13B/p‐STAT3 Axis

**DOI:** 10.1002/advs.202409416

**Published:** 2024-12-25

**Authors:** Hongmei Zheng, Yaoxiang Tang, Hongjing Zang, Jiadi Luo, Hanqiong Zhou, Yuting Zhan, Ying Zou, Qiuyuan Wen, Jian Ma, Songqing Fan

**Affiliations:** ^1^ Department of Pathology The Second Xiangya Hospital Central South University Changsha Hunan 410011 China; ^2^ Hunan Clinical Medical Research Center for Cancer Pathogenic Genes Testing and Diagnosis Changsha Hunan 410011 China; ^3^ Cancer Research Institute of Central South University Changsha Hunan 410078 China

**Keywords:** DUSP13B, itraconazole, osimertinib resistance, p‐STAT3, SHH

## Abstract

There is an urgent necessity to devise efficient tactics to tackle the inevitable development of resistance to osimertinib, which is a third‐generation epidermal growth factor receptor (EGFR) inhibitor used in treating EGFR‐mutant nonsmall cell lung cancer (NSCLC). This study demonstrates that combining itraconazole with osimertinib synergistically reduces the proliferation and migration, enhances the apoptosis of osimertinib‐resistant cells, and effectively inhibits the growth of osimertinib‐resistant tumors. Mechanistically, itraconazole combined with osimertinib promotes the proteasomal degradation of sonic hedgehog (SHH), resulting in inactivation of the SHH/Dual‐specificity phosphatase 13B (DUSP13B)/p‐STAT3 and Hedgehog pathways, suppressing Myc proto‐oncogene protein (c‐Myc). Additionally, DUSP13B interacts with signal transducer and activator of transcription 3 (STAT3) and modulates its phosphorylation. Interestingly, it is observed that SHH overexpression partially rescues the synergistic effects of this combination treatment strategy through the SHH/DUSP13B/p‐STAT3 signaling axis. Moreover, it is found that SHH, (GLI1), p‐STAT3, and DUSP13B play significant predictive roles in osimertinib resistance. In lung adenocarcinoma, p‐STAT3 is positively correlated with SHH but negatively correlated with DUSP13B. Together, these results highlight the crucial role of itraconazole in reversing the acquired resistance to osimertinib and provide a scientific rationale for the therapeutic strategy of combining osimertinib with itraconazole.

## Graphical abstract

Itraconazole directly binds to sonic hedgehog (SHH) and promotes its ubiquitin‐mediated degradation, which results in the upregulation of DUSP13B and dephosphorylation of STAT3. This inactivates the SHH/DUSP13B/p‐STAT3 axis and the Hedgehog pathway, leading to the suppression of c‐Myc and contributing to the reversal of osimertinib resistance.

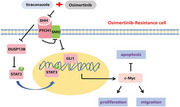

## 1 Introduction

Lung cancer constitutes 11.4% of the total cancer incidence and 18.0% of global cancer‐related deaths.^[^ ^1^ ^]^ The increased application of low‐dose computed tomography screening has improved the early detection rates of lung cancer, leading to a decrease in mortality. However, most patients are diagnosed with distant metastases, and the efficacy of traditional treatment modalities remains unsatisfactory.^[^ ^2^, ^3^ ^]^ Among Asian populations, EGFR gene mutations are the most prevalent driver mutations in lung cancer, with mutation rates ranging from 30% to 50%.^[^ ^4^ ^]^ EGFR‐TKIs are small‐molecule drugs that specifically target mutations in the EGFR gene. They competitively bind to the ATP‐binding site of EGFR, inhibiting EGFR autophosphorylation, thereby blocking signal transduction and exerting antitumor effects.^[^ ^5^ ^]^ Following treatment with first‐ and second‐generation EGFR‐TKIs, the majority of patients ultimately develop a T790M resistance mutation. Osimertinib, a third‐generation EGFR‐TKI, can overcome this resistance, significantly prolonging the survival of patients with EGFR‐mutant lung cancer. However, owing to tumor heterogeneity and the pressure of drug selection, patients treated with osimertinib ultimately develop resistance.^[^ ^6^, ^7^ ^]^

The mechanisms leading to osimertinib resistance mainly fall into two categories: EGFR‐dependent and EGFR‐independent.^[^ ^8^ ^]^ EGFR‐independent mechanisms mainly involve the activation of alternative pathways (such as MET amplification, activation of the phosphatidylinositol 3‐kinase (PI3K) and RAS‐mitogen‐activated protein kinases (MAPK) pathway, and overexpression of AXL), and histological transformation.^[^ ^8^ ^]^ Although some progress has been made in understanding osimertinib resistance, owing to its heterogeneity and complexity, we are still far from fully elucidating its complete mechanisms, and solutions to overcome osimertinib resistance have not yet been found. Therefore, more research is needed to explore the mechanisms of acquired resistance to osimertinib and find solutions to overcome it, ultimately improving the prognosis of patients with lung cancer.

Drug repurposing is an important strategy in drug development, as it significantly reduces the cost of drug research by identifying drugs from existing clinical therapies that can reverse resistance to osimertinib. Preliminary screening revealed that itraconazole inhibited the proliferation of osimertinib‐resistant cells. Itraconazole is a widely used antifungal drug in clinical practice. Recently, several studies have indicated that itraconazole not only exhibits antifungal activity but also exerts inhibitory effects on various malignancies, including hepatocellular carcinoma, basal cell carcinoma, prostate cancer, and melanoma.^[^ ^9^ ^]^ Itraconazole exerts antitumor effects via multiple pathways. Studies have demonstrated that itraconazole can inhibit the growth of liver cancer cells and promote apoptosis through the AKT/mTOR/S6K, reactive oxygen species (ROS), and Wnt/β‐catenin pathways, thereby exerting anticancer effects.^[^ ^10^ ^]^ In esophageal cancer, itraconazole acts by inhibiting the HER2/AKT signaling pathway.^[^ ^11^ ^]^ Although itraconazole exerts potent antitumor effects, its specific molecular mechanisms are not fully understood. To date, no studies have reported the potential application of itraconazole in overcoming acquired resistance to osimertinib.

The Hh pathway is highly conserved and is primarily composed of ligands, receptors, transcription factors, and downstream target genes. Mammals have three types of ligands: Indian Hedgehog (IHH), Desert Hedgehog (DHH), and Sonic Hedgehog (SHH). Among them, SHH is the most important. When the SHH ligand is present, it binds to the receptor protein PTCH1, leading to the translocation of the transcription factor GLI into the nucleus and initiating the transcription of downstream target genes.^[^ ^12^, ^13^ ^]^ Recently, studies have found that dysregulated activation of the Hh signaling pathway correlates with drug resistance. Research has found that high SHH expression is related to a poor objective response rate to EGFR‐TKIs and shorter progression‐free survival.^[^ ^14^ ^]^ Furthermore, studies have found that using smoothened (SMO) inhibitors to block the hedgehog pathway restores the sensitivity of resistant cells to EGFR‐TKIs.^[^ ^15^ ^]^

Signal transducer and activator of transcription 3 (STAT3) belongs to the STAT family of proteins and is activated by the Janus kinase (JAK)/STAT pathway. JAK is a tyrosine kinase protein that is activated when cytokines bind to surface receptors, leading to the phosphorylation and activation of STAT3. Activated STAT3 enters the nucleus in the form of dimers and transcribes downstream target genes.^[^ ^16^ ^]^ Research has shown that STAT3 activation can also occur through JAK‐independent mechanisms, such as phosphorylation of the Tyr705 site by pyruvate kinase M2.^[^ ^17^ ^]^ In the past several years, studies have found that p‐STAT3 is associated with EGFR‐TKI resistance, and inhibition of the p‐STAT3 can enhance the sensitivity of lung cancer cells to EGFR‐TKIs.^[^ ^18^ ^]^

Dual‐specificity phosphatase 13B (DUSP13B) belongs to the DUSPs protein family and can dephosphorylate phosphorylated tyrosine and serine/threonine residues.^[^ ^19^ ^]^ Research has suggested that the coexpression of DUSP13B and DUSP4 weakens transforming growth factor β‐1 (TGFβ‐1)‐mediated invasion, migration, and chemotherapy resistance in lung cancer.^[^ ^20^ ^]^ However, there have been no investigations into the involvement of DUSP13B in osimertinib resistance.

Autophagy is a process of self‐degradation and recycling of cellular components, and is essential for maintaining cellular homeostasis and survival. It is typically classified into macroautophagy, chaperone‐mediated autophagy, and microautophagy.^[^ ^21^, ^22^ ^]^ Autophagy plays a crucial role in cancer progression, metastasis, and drug resistance. First, it can serve as a protective mechanism leading to resistance to chemotherapy. It has been shown that autophagy is involved in cisplatin resistance in lung cancer cells, and autophagy inhibitors can enhance sensitivity to cisplatin.^[^ ^23^ ^]^ In addition, chemotherapeutic drugs can also induce autophagic cell death. It has been found that esomeprazole inhibits the proliferation of paclitaxel‐resistant cells by inducing autophagy.^[^ ^24^ ^]^ Additionally, autophagy has been implicated in the resistance to EGFR‐TKIs. Research has revealed that an HIF‐1α inhibitor enhances gefitinib‐induced apoptosis by inhibiting autophagy.^[^ ^25^ ^]^ Due to the dual nature of autophagy, it is essential to explore whether autophagy is induced and its role in the therapeutic process of anticancer drugs to fully enhance anticancer efficacy.

This study aimed to explore the role of itraconazole in overcoming acquired resistance to osimertinib and its specific molecular mechanisms, thereby providing new perspectives and ideas for overcoming osimertinib resistance.

## 2 Experimental Section

### 2.1 Reagents and Cell Lines

Osimertinib was purchased from Selleck (Houston, TX). Itraconazole, chloroquine, MG132, and CHX were obtained from MedChemExpress (Monmouth Junction, NJ). All drugs were dissolved in Dimethyl sulfoxide (DMSO). The complete list of antibodies is provided in Table S1 (Supporting Information). SHH protein was purchased from SinoBiological (Beijing, China). Immunoprecipitation Assay Kit was purchased from Beyotime (Shanghai, China). The Annexin V‐FITC/7AAD Apoptosis Detection Kit was procured from MultiSciences (Hangzhou, China). The PC‐9/AR (osimertinib‐resistant cell line) was generously gifted by Prof. Shi‐Yong Sun (Emory University, USA). HCC827/AR cells were constructed using a concentration gradient dependency. PC‐9 and HCC827 are osimertinib‐sensitive cell lines.

### 2.2 Cell Counting Kit‐8 (CCK‐8)

Cells were plated at an appropriate density in a 96 well plate, and drug treatment (either alone or in combination) was commenced the following day. Cell viability was evaluated employing the Cell Counting Kit‐8 (CCK‐8). The combination index (CI) for drug interactions was estimated using the CompuSyn software. A CI value greater than 1 is indicative of an antagonistic relationship. A CI value less than 1 signifies a synergistic effect and a CI value equal to 1 represents an additive effect.

### 2.3 Colony Formation Assays

Cells were plated at an appropriate density in a 6 well plate, and drug treatment (either alone or in combination) was commenced the following day for 9–12 days. The drug‐containing medium was refreshed at regular intervals of 2–3 days. Subsequently, the colonies underwent a fixation process utilizing 4% paraformaldehyde, followed by a staining procedure with crystal violet for a duration of 20 min. Finally, colonies were photographed and counted.

### 2.4 Apoptosis Assay and Cell Cycle Analysis

Cells were collected, washed with PBS, and detected using the Apoptosis Detection Kit. The apoptosis rate was examined using the FlowJo V10 software. Cell cycle analysis was performed using a reagent kit and analyzed by Modifit software.

### 2.5 Western Blotting

After treating the cells with various methods, they were lysed, and the protein supernatant was collected. Protein samples underwent electrophoretic separation via Sodium Dodecyl Sulfate Polyacrylamide Gel Electrophoresis (SDS‐PAGE) and were subsequently transferred onto a Polyvinylidene Fluoride (PVDF) membrane for further analysis. After blocking with a 5% skim milk solution at ambient temperature for a duration of 2 h, the membrane underwent incubation with the primary antibody at 4 °C for an overnight period. Following the primary antibody incubation, the membrane was subjected to a secondary antibody incubation step at 37 °C for a period of 1 h and detected using an enhancedchemiluminescence (ECL) system.

### 2.6 Animal Experiments

Female BALB/c nude mice were housed in a pathogen‐free facility. PC‐9/AR cells were injected subcutaneously into nude mice. Once the tumor volume reached between 50 and 100 mm^3^, female BALB/c nude mice were randomly assigned to four distinct experimental groups, each consisting of six mice: a vehicle group, an itraconazole alone group (75 mg kg^−1^ day^−1^), an osimertinib alone group (5 mg kg^−1^ day^−1^), and a combination therapy group. Tumor volume and body mass were measured at a frequency of every 3 days. The tumor volume was determined as follows: V = length × (width^2^)/2. After excision, the tumors were weighed and subsequently immersed in 10% neutral buffered formalin for fixation. The experimental procedures involving animals were authorized by the Animal Welfare and Ethics Review Committee of Hunan Yuantai Biotechnology (YT‐T‐20211209‐1) and complied with the ethical regulations.

### 2.7 Immunohistochemistry (IHC) Staining

IHC staining of human NSCLC tissue specimens and nude mouse tumor tissue specimens was conducted following a previously described protocol.^[^ ^26^, ^27^ ^]^ The semiquantitative evaluation method was as follows: total score = intensity score×percentage score. The intensity scores encompassed four levels: 0 (negative), 1 (weak), 2 (moderate), and 3 (strong), while the extent of staining was categorized based on the proportion of stained cells: 0 (no staining), 1 (1%–25%), 2 (26%–50%), 3 (51%–75%), and 4 (76%–100%).

### 2.8 Lentivirus Infection

Cells were seeded into 24 well plate at an appropriate density and then infected with the viruses LV‐SHH, LV‐DUSP13B, and the control virus LV‐NC. Following a 48‐h infection period, cells underwent puromycin screening (2–5 µg µL^−1^) to establish stable cell lines.

### 2.9 Quantitative PCR Analysis

Total RNA extraction was performed using the TRIzol reagent (Invitrogen). Reverse transcription and quantitative reverse transcription PCR were executed according to the Takara protocol. Primers used are listed in Table S2 (Supporting Information).

### 2.10 Isothermal Titration Calorimetry (ITC) Measurement

The ITC experiments were conducted at 25 °C. First, itraconazole and SHH protein were diluted in phosphate‐buffered saline (PBS) containing 5% DMSO to final concentrations of 50 and 5 µm, respectively. The SHH protein was added to the cells and titrated with 50 µm itraconazole. Dispense 2.5 µL of itraconazole for each titration, with a total of 20 titrations. Data analysis was used NanoAnalyze Software.

### 2.11 Coinmunoprecipitation (Co‐IP)

The cellular lysate was subjected to an overnight incubation at 4 °C with 3 µg each of DUSP13B and STAT3 antibodies, along with a negative control IgG antibody. After adding Protein A/G beads, the resulting mixture was further incubated for 3 h at 4 °C. After washing the beads to remove nonspecific interactions, the tightly bound proteins were eluted and subsequently heated to 100 °C for 10 min.

### 2.12 Immunofluorescence (IF)

The cells were subjected to a 30‐min fixation protocol at 37 °C using 4% paraformaldehyde, followed by permeabilization with 0.25% Triton X‐100 for 30 min. Subsequently, they were blocked with 5% BSA for 1 h and then incubated with the primary antibody overnight at 4 °C. Next day, the cells were incubated with fluorescent secondary antibody at 37 °C for 1 h and DAPI for 5 min.

### 2.13 Bioinformatics Databases and Clinical Samples

The dataset GSE193258 was sourced from the GEO database, focusing on osimertinib resistance and comprising four cell lines with EGFR mutations (PC9, HCC827, H1975, and HCC2935). R software was used for bioinformatic analyses. The relationship between the mRNA expression levels of different genes and EGFR gene mutation status was assessed using the TIMER2.0 website (http://timer.cistrome.org/). Additionally, the association between the mRNA expression levels of various genes was investigated using the GEPIA2 website.

Moreover, this study collected ten pairs of lung cancer tissue specimens before and after osimertinib resistance from the pathology department of the Second Xiangya Hospital of Central South University. Additionally, tumor tissue were obtained from 108 patients with lung adenocarcinoma who underwent surgical treatment, along with clinicopathological characteristics and prognosis data (**Table** **1**). This study was approved by the Scientific Research Ethics Committee.

**Table 1:** Clinicopathological features of 108 cases of lung adenocarcinoma.

Clinicopathological features	N [%]
Gender	
Female	61 (56.5)
Male	47 (43.5)
Age (years)	
<60	62 (57.4)
≥60	46 (42.6)
Pathological degree	
Well and moderated differentiation	72 (66.7)
Poor differentiation	36 (33.3)
EGFR mutation status	
Mutant‐Type	80 (74.1)
Wild‐Type	28 (25.9)
Clinical stages	
Stage I	47 (43.5)
Stage II and III	61 (56.5)
Lymph node metastasis	
Yes	40 (37.0)
No	68 (63.0)
Smoking history	
Yes	27 (25.0)
No	81 (75.0)

### 2.14 Transmission Electron Microscope (TEM)

The cells were harvested and then treated with a 2.5% glutaraldehyde solution for fixation at 4 °C for 4–6 h. Subsequently, the samples were sent to an electron microscopy room for further processing. Transmission electron microscopy was used to capture digital images.

### 2.15 Statistical Analysis

Data processing and analysis were conducted using GraphPad Prism version 8.0, SPSS 24.0, and R 4.2.1. Statistical differences were accomplished using unpaired Student's *t*‐tests and ANOVA test. The Wilcoxon signed‐rank test was employed to analyze the expression levels of different proteins in lung cancer tissues before and after osimertinib resistance. To assess the correlation between various proteins, the Spearman's coefficient was applied. Data were presented as mean ± SD and obtained from at least three independent experiments. Statistical significance was defined as *P* < 0.05.

## 3 Results

### 3.1 Itraconazole Combined with Osimertinib had a Synergistic Effect on Osimertinib‐Resistant NSCLC Cells In Vitro

**Figure 1:**
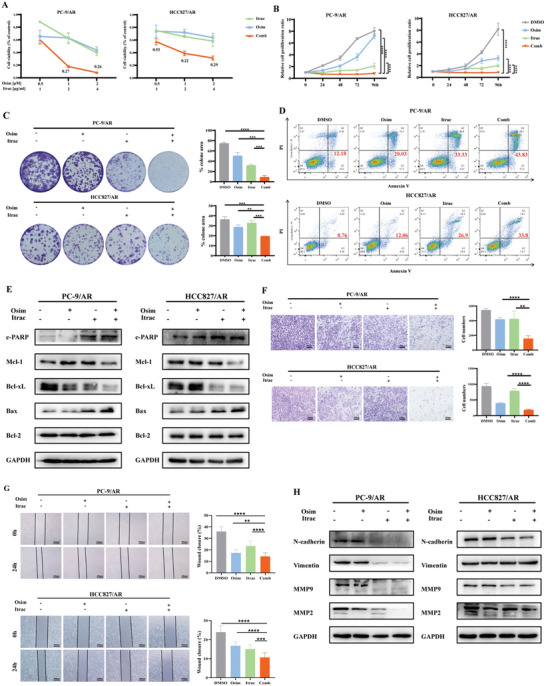
Itraconazole and osimertinib had a synergistic effect in vitro. A) CI of itraconazole in combination with osimertinib in PC‐9/AR and HCC827/AR cells. The numbers represented the CI values, which were calculated using CompuSyn software. B,C) The effect of itraconazole in combination with osimertinib on the proliferation of PC‐9/AR and HCC827/AR cells was determined by CCK8 and colony formation. D) Itraconazole combined with osimertinib synergistically promoted apoptosis of PC‐9/AR and HCC827/AR cells examined by flow cytometry. E) The expression of apoptosis‐related proteins was detected by western blotting. F,G) Itraconazole in combination with osimertinib synergistically inhibited migration demonstrated by wound healing assay and transwell assay. H) The expression of migratory invasion‐associated proteins was detected by western blotting. Osim: osimertinib; Itrac: itraconazole; Comb: the combination of osimertinib and itraconazole. Data were presented as mean ± SD (*n* = 3), and the *P* value was calculated using unpaired student's *t*‐tests and two‐way ANOVA test. **P < 0.01; ***P < 0.001; ****P < 0.0001.

Drug repurposing is an important strategy in drug development, as it significantly reduces the cost of drug research by identifying drugs from existing clinical drugs that can reverse resistance to osimertinib. Preliminary screening revealed the ability of itraconazole to inhibit the proliferation of osimertinib‐resistant cells (Figure S1, Supporting Information). To further elucidate whether the combination of itraconazole and osimertinib demonstrates a synergistic antitumor effect, we treated osimertinib‐resistant cells with varying concentrations of these two drugs in combination and assessed cell proliferation activity after 48 h. We then calculated the Combination Index (CI) and found that the CI values were <1 when itraconazole was combined with osimertinib in HCC827/AR and PC‐9/AR cells, signifying their synergistic antitumor effects (**Figure** **1**A). Moreover, itraconazole augmented the osimertinib's ability to suppress cell proliferation and colony formation (Figure 1B,C). We evaluated the levels of apoptosis and observed that itraconazole enhanced the apoptotic effect of osimertinib (Figure 1D). Additionally, we also investigated the expression of apoptosis‐associated proteins and observed that itraconazole in combination with osimertinib promoted the expression of Bax and cleaved PARP, while suppressing the expression of Bcl‐xL and Mcl‐1, both recognized as antiapoptotic proteins (Figure 1E). Furthermore, we assessed the effect of the combination therapy on the cell cycle and noted that the combination treatment synergistically induced G0/G1 phase arrest (Figure S2, Supporting Information). Interestingly, we found that combination therapy synergistically inhibited the migration ability of osimertinib‐resistant cells, as evidenced by the decreased levels of MMP9, MMP2, and N‐cadherin proteins (Figure 1F–H).

### 3.2 Itraconazole in Combination with Osimertinib Exerted its Effects Through the SHH/DUSP13B/p‐STAT3 Axis

**Figure 2:**
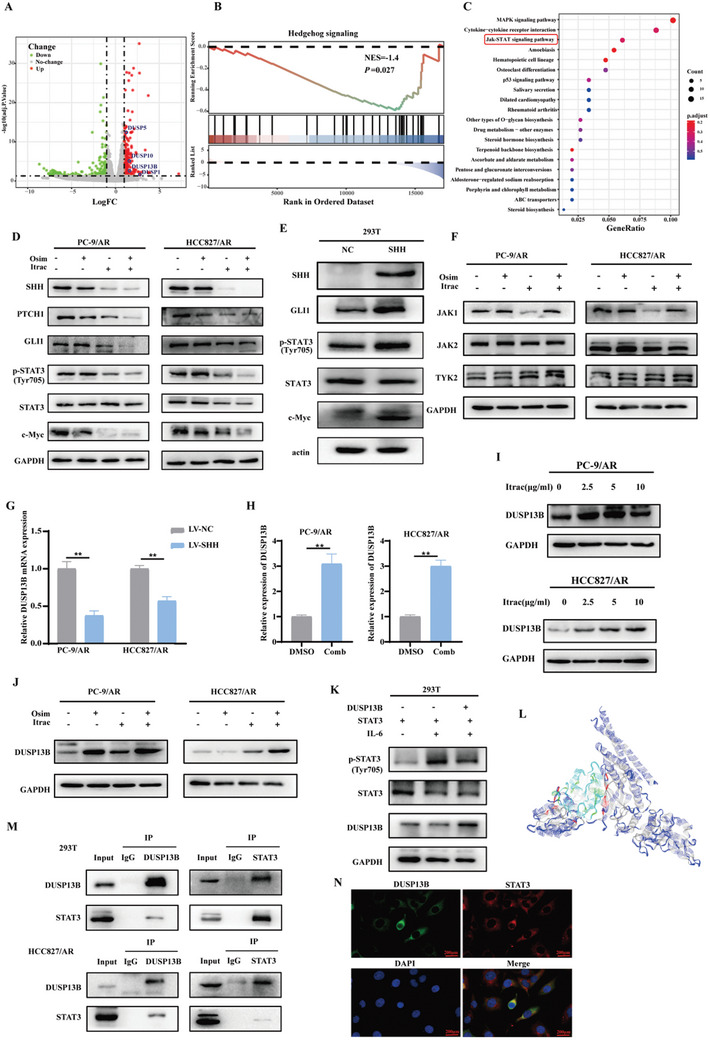
Itraconazole combined with osimertinib exerted effects through the SHH/DUSP13B/p‐STAT3 axis. A) Volcano plot of differential gene expression before and after itraconazole treatment. B,C) Potential molecular mechanisms were identified through KEGG and GSEA analysis. D) Itraconazole combined with osimertinib modulated the key proteins of Hedgehog and STAT3 signaling pathway. E) SHH regulated the level of STAT3 phosphorylation. F) Regulation of JAKs proteins was detected by western blotting. G) The mRNA expression of DUSP13B after SHH overexpression was detected by qPCR. LV‐NC: the control group; LV‐SHH: stable overexpression of the SHH group. H–J) The mRNA and protein expression of DUSP13B after the combination of itraconazole with osimertinib. K) DUSP13B exhibited a dephosphorylation effect on STAT3, as confirmed by in vivo phosphorylation experiments. L) The result of molecular docking between STAT3 and DUSP13B. Blue represented STAT3 and green represented DUSP13B. M) Co‐IP demonstrated the binding of DUSP13B to STAT3. N) IF confirmed the colocalization between DUSP13B and STAT3 in HCC827/AR cells. DUSP13B is labeled in green, STAT3 in red, and the nucleus in blue. Osim: osimertinib; Itrac: itraconazole; Comb: the combination of osimertinib and itraconazole. Data were presented as mean ± SD (*n* = 3), and the *P* value was calculated using unpaired student's *t*‐tests. **P < 0.01.

To gain insights into the specific molecular mechanism by which itraconazole reverses the acquired resistance to osimertinib, we conducted RNA‐seq on PC‐9/AR cells treated with itraconazole or DMSO. A volcano plot of the differentially expressed genes was depicted in **Figure** **2**A. GSEA and KEGG pathway enrichment analysis suggested that itraconazole might target the Hedgehog and JAK‐STAT signaling pathways (Figure 2B,C). Therefore, we evaluated the expression of key proteins in these pathways, such as SHH, PTCH1, GLI1, STAT3, p‐STAT3, and its downstream target protein c‐Myc, using western blotting, both in the presence of itraconazole alone and in combination with osimertinib (Figure S3 (Supporting Information) and Figure 2D). We also found that the mRNA expression of SHH was slightly reduced after treatment with itraconazole (Figure S3, Supporting Information). In order to gain a thorough understanding of the potential interactions between the Hh and STAT3 pathways, we overexpressed the SHH plasmid in 293T cells and subsequently assessed the key proteins of these two signaling pathways. The results (Figure 2E) indicated a notable elevation in the expression of p‐STAT3 (Tyr705) and c‐Myc after SHH overexpression, suggesting a potential interaction between the Hh pathway and the STAT3 pathway via the SHH‐p‐STAT3 axis.

Based on these findings, we initially confirmed the existence of the SHH‐p‐STAT3 signaling axis. As SHH has not been reported to have kinase or phosphatase activity, we speculated that the regulation of the SHH‐p‐STAT3 signaling axis by the combination of itraconazole and osimertinib might involve other kinases or phosphatases. First, we examined the expression levels of the most common kinases in the STAT3 pathway and found no significant changes in the JAK1, JAK2, and TYK2 proteins (Figure 2F). It has been shown that dual‐specificity phosphatases (DUSPs) play a crucial role in cellular physiology by dephosphorylating residues of phosphoserine, phosphothreonine, and phosphotyrosine.^[^ ^28^ ^]^ Since the Tyr705 site of p‐STAT3 is a phosphorylated tyrosine, we further focused our attention on DUSPs. As shown in Figure 2A, DUSP1, DUSP5, DUSP10, and DUSP13B levels were significantly increased after treatment with itraconazole. Thus, we conducted further analysis to examine the correlation between these DUSPs and SHH and found a significant negative correlation between DUSP13B and SHH (Figure S4, Supporting Information). Furthermore, we noted a substantial reduction in the mRNA expression of DUSP13B after SHH overexpression (Figure 2G). Therefore, we hypothesized that the combination of itraconazole and osimertinib most likely induces dephosphorylation of p‐STAT3 by upregulating DUSP13B. Indeed, both the mRNA and protein levels of DUSP13B were markedly elevated after combination therapy (Figure 2H–J).

### 3.3 DUSP13B Interacted with STAT3 and Dephosphorylated STAT3

To further validate whether DUSP13B can dephosphorylate p‐STAT3, we conducted in vivo phosphorylation experiments and found that DUSP13B had a dephosphorylation effect on p‐STAT3 (Figure 2K). Subsequently, we investigated whether DUSP13B achieves dephosphorylation by directly binding to STAT3. Initially, we performed molecular docking analysis of STAT3 and DUSP13B using the ZDOCK online website. The docking result revealed that the docking binding free energy ΔiG was <0, indicating that the docking was stable (Figure 2L). To further validate the physical interaction between DUSP13B and STAT3, we conducted co‐IP and immunofluorescence experiments. These experiments confirmed that DUSP13B interacted with STAT3 (Figure 2M,N).

### 3.4 Overexpression of DUSP13B Reversed Osimertinib Resistance in NSCLC

As there have been no reports on the involvement of DUSP13B in acquired resistance to osimertinib, we investigated the role of DUSP13B in osimertinib‐acquired resistance. Remarkably, we observed a significant reduction in the mRNA and protein expression levels of DUSP13B in both PC‐9/AR and HCC827/AR cells (Figure S5A,B, Supporting Information). Based on these findings, we proceeded to construct DUSP13B overexpressing stable cell lines. Intriguingly, we found that overexpressing DUSP13B resulted in a reduction of the IC50 value osimertinib‐resistant cells (Figure S5C,D, Supporting Information). Furthermore, our investigations revealed that overexpression of DUSP13B not only inhibited the proliferation and migration of osimertinib‐resistant cells but also promoted apoptosis (Figure S5E–G, Supporting Information). Taken together, these findings strongly indicate that overexpression of DUSP13B could be a promising approach to overcome osimertinib resistance in NSCLC.

### 3.5 Overexpression of SHH Rescued the Synergistic Effect of Itraconazole and Osimertinib

**Figure 3:**
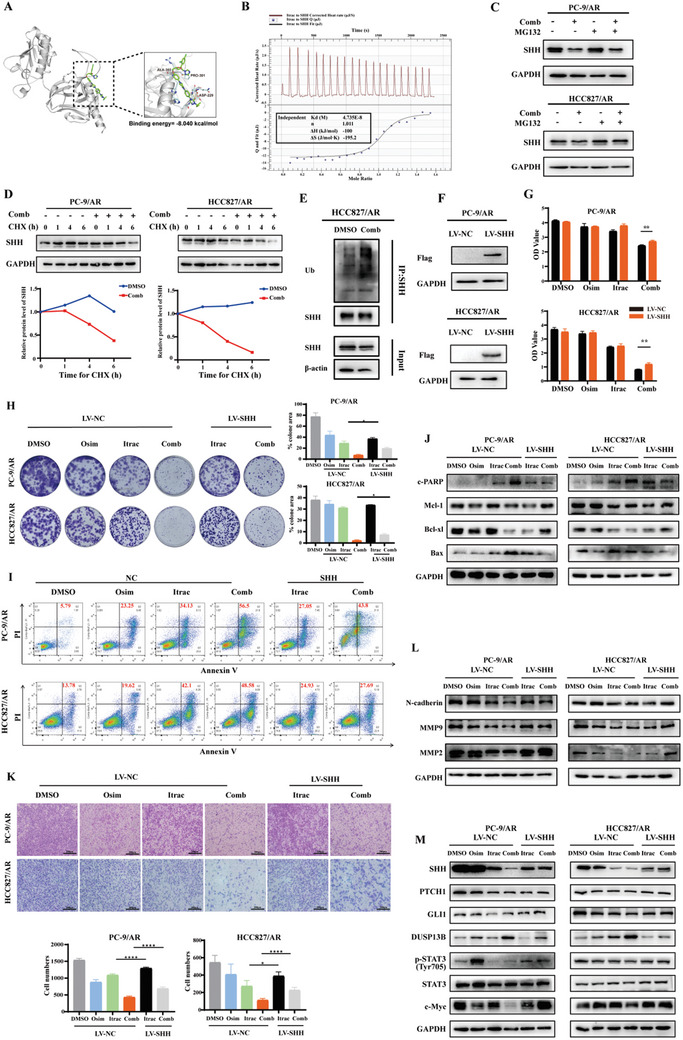
Overexpression of SHH rescued the synergistic effect of itraconazole and osimertinib. A) The molecular docking schematic of itraconazole and SHH. B) Isothermal titration calorimetry was used to measure the binding between itraconazole and STAT3. C) MG132 abolished the ability of itraconazole combined with osimertinib to decrease SHH level. D) Itraconazole combined with osimertinib shortened the half‐life of SHH protein. E) The HCC827/AR cells were treated with combination therapy for 36 h, followed by treatment with MG132 for 6 h before harvesting. Ubiquitination levels were then assessed after immunoprecipitation with SHH antibody. F) Successful establishment of stable cell lines overexpressing SHH was validated by western blotting. G,H) Overexpression of SHH partially rescued the synergistic antiproliferative effect of itraconazole combined with osimertinib by CCK8 and colony formation assay. I) Overexpression of SHH partially rescued the synergistic proapoptotic ability of itraconazole combined with osimertinib. J) The expression of apoptosis‐related proteins was detected by western blotting. K) Overexpression of SHH partially rescued the synergistic antimigratory capacity of itraconazole combined with osimertinib. L) The expression of migratory invasion‐associated proteins was detected by western blotting. M) Overexpression of SHH could partly reverse the regulation of itraconazole combined with osimertinib on SHH/DUSP13B/p‐STAT3 signaling axis. Osim: osimertinib; Itrac: itraconazole; Comb: the combination of osimertinib and itraconazole; CHX: Cycloheximide. Data were presented as mean ± SD (*n* = 3), and the *P* value was calculated using unpaired student's *t*‐tests. *P < 0.05; **P < 0.01; ****P < 0.0001.

We further elucidated the mechanism of action underlying the combination of itraconazole and osimertinib in reducing SHH protein expression levels. We initially used AutoDock software for molecular docking simulations of Itraconazole and SHH, and found that itraconazole had the capability to bind to SHH (**Figure** **3**A). So, we used ITC to further examine the interaction between SHH and itraconazole and found that there was a direct binding between itraconazole and SHH (Figure 3B). We then used MG132 to treat osimertinib‐resistant cells and observed that MG132 could restore the reducing effect of the combination therapy on SHH protein (Figure 3C). Furthermore, we conducted CHX assays and found that SHH degradation was faster in osimertinib‐resistant cells exposed to a combination of itraconazole and osimertinib than in the DMSO group, indicating that the combination therapy facilitated the degradation of SHH (Figure 3D). To further confirm that combination therapy promotes the ubiquitin‐mediated degradation of SHH protein, we measured the levels of polyubiquitinated SHH in osimertinib‐resistant cells. As shown in Figure 3E, combination therapy led to a significant increase in SHH polyubiquitination. In summary, these results indicated that combination therapy induced SHH ubiquitin‐mediated degradation.

Previous findings have suggested that itraconazole in combination with osimertinib exerts its effects through the SHH/DUSP13B/p‐STAT3 signaling axis. To verify whether the overexpression of SHH could rescue the synergistic effect of itraconazole and osimertinib, we constructed stable cell lines overexpressing SHH, which were validated by western blotting (Figure 3F). As expected, overexpression of SHH partially rescued the synergistic effects of itraconazole and osimertinib, including proliferation, apoptosis, and migration (Figure 3G–L). In addition, we observed that overexpression of SHH could partly reverse the regulation of itraconazole combined with osimertinib on the SHH/DUSP13B/p‐STAT3 signaling axis (Figure 3M).

### 3.6 The Combination of Itraconazole and Osimertinib Induced Autophagy in Osimertinib‐Resistant Cells

**Figure 4:**
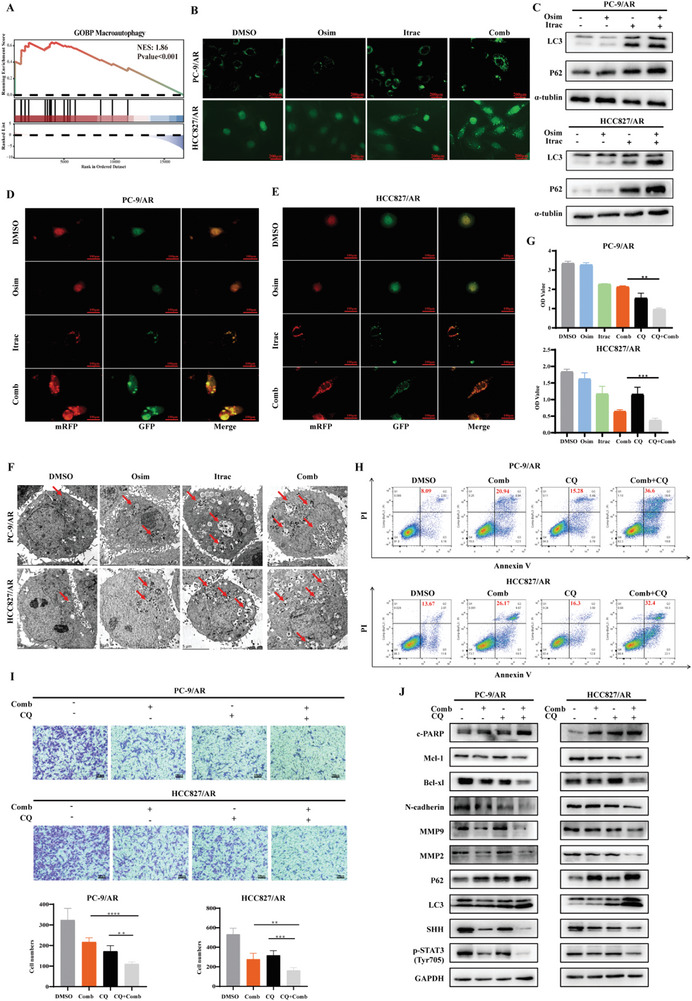
The combination of itraconazole and osimertinib induced autophagy in osimertinib‐resistant cells. A) GSEA analysis showed that autophagy was enriched in the itraconazole‐treated group. B) IF demonstrated the impact of itraconazole combined with osimertinib on LC3. C) The expression of autophagy‐related proteins was detected by western blotting. D,E) Autophagic flux was analyzed by mRFP‐GFP‐LC3. Red dots represented autolysosomes, and yellow dots represented autophagosomes. F) Observation of intracellular autophagosomes by TEM. G) CQ further inhibited proliferation detected by CCK8 assay. H) CQ further increased apoptosis as detected by flow cytometry I) CQ further suppressed the migratory capacity. J) The indicated proteins were detected using western blotting. Osim: osimertinib; Itrac: itraconazole; Comb: the combination of osimertinib and itraconazole; CQ: chloroquine. Data were presented as mean ± SD (*n* = 3), and the *P* value was calculated using unpaired student's *t*‐tests. **P < 0.01; ***P < 0.001; ****P < 0.0001.

When analyzing the RNA‐seq results, GSEA enrichment analysis suggested that itraconazole can induce autophagy in PC‐9/AR cells (**Figure** **4**A). Therefore, we explored the effect of itraconazole combined with osimertinib on autophagy. As depicted in Figure 4B, we observed a substantial increase in granular LC3, indicating an increase in autophagosomes in the combination therapy group. Additionally, the ratio of LC3II/LC3I and P62 were also significantly increased (Figure 4C). mRFP‐GFP‐LC3 was used for real‐time detection of autophagy flux, with GFP (green) and RFP (red) tagged to LC3. The green fluorescence of GFP on autolysosomes was denatured in an acidic environment, resulting in only red fluorescence inside the cells, whereas the autophagosomes displayed yellow fluorescence. We noticed a substantial rise in the number of yellow dots (autophagosomes) and red dots (autolysosomes) in the combination therapy group, suggesting an increase in autophagic flux (Figure 4D–E). TEM showed that itraconazole combined with osimertinib significantly increased the count of autophagosomes in osimertinib‐resistant cells (Figure 4F).

As mentioned above, the combination of itraconazole and osimertinib promotes autophagy in osimertinib‐resistant cells. To clarify the specific role of autophagy, we used chloroquine (CQ) to inhibit autophagy by suppressing the fusion and degradation of autolysosomes.^[^ ^29^ ^]^ Compared with the combination drug group, CQ combined with itraconazole and osimertinib further inhibited the growth of osimertinib‐resistant cells, as shown by the CCK8 assay (Figure 4G). Analysis of apoptosis (Figure 4H) revealed that the synergistic proapoptotic effect of itraconazole in combination with osimertinib was further enhanced after inhibiting autophagy using CQ. Moreover, the results indicated that CQ further enhanced the synergistic antimigratory effect of the combination of itraconazole and osimertinib (Figure 4I). We performed western blotting experiments and found that after inhibiting autophagy, the regulatory effects of itraconazole combined with osimertinib on apoptosis‐ and migration‐related proteins were further enhanced. Unexpectedly, we found that the levels of p‐STAT3 and SHH were further reduced after inhibiting autophagy (Figure 4J).

### 3.7 Itraconazole and Osimertinib had a Synergistic Effect In Vivo

**Figure 5:**
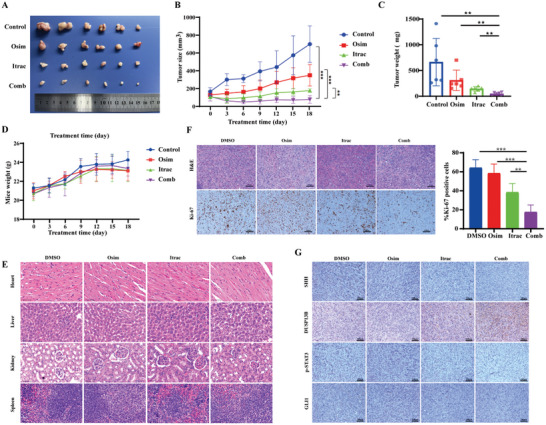
The combination of itraconazole and osimertinib effectively inhibited the growth of osimertinib‐resistant tumors in vivo. A) Subcutaneous tumors were excised from nude mice, and the tumor size was observed and photographed. B) Tumor size was measured and calculated as, V = length × (width^2^)/2. C) Differences in tumor weight of subcutaneously transplanted tumors. D) Changes in body weight among four groups of nude mice. E) H&E staining of the heart, liver, kidney, and spleen of nude mice. F) Representative images of H&E and Ki‐67 staining. G) Representative images of IHC staining of SHH/DUSP13B/p‐STAT3 axis. Osim: osimertinib; Itrac: itraconazole; Comb: the combination of osimertinib and itraconazole. Data were presented as mean ± SD (*n* = 6), and the *P* value was calculated using unpaired Student's *t*‐tests and two‐way ANOVA test. **P < 0.01; ***P < 0.001.

Furthermore, to clarify whether the combination of itraconazole and osimertinib exhibits synergistic effects in vivo and to establish a robust theoretical basis for its clinical application, we conducted preliminary validation of the therapeutic efficacy and dosing safety of the combination therapy in vivo. The results (**Figure** **5**A,B) revealed a significant decrease in tumor volume within the combination group compared to the other groups. Additionally, the combination of itraconazole and osimertinib markedly reduced the tumor weight (Figure 5C). The growth curves of the nude mice were shown in Figure 5D. Moreover, we conducted H&E staining of the liver, heart, kidney, and spleen of nude mice and observed no damage to these organs across the four groups, indicating that the combination of itraconazole and osimertinib did not result in significant toxic side effects (Figure 5E). We further conducted H&E and Ki‐67 staining of the subcutaneous tumor tissues of mice. As shown in Figure 5F, the Ki‐67 expression level was the lowest in the combination group, indicating that the combination significantly reduced the proliferative capacity of tumor tissues. To investigate whether itraconazole combined with osimertinib can suppress the expression of the SHH/DUSP13B/p‐STAT3 signaling axis in vivo, IHC staining was performed. As expected, the expression of SHH, p‐STAT3, and GLI1 was markedly decreased in the combination group, whereas DUSP13B expression was significantly increased, which was consistent with our in vitro findings (Figure 5G). In addition, we also validated the effect of combined treatment on the autophagy protein LC3 in vivo, and found that the combined treatment group had the highest expression of LC3 protein (Figure S6, Supporting Information).

### 3.8 The Expression of SHH/DUSP13B/p‐STAT3 in Lung Adenocarcinoma Tissues and their Correlation with Osimertinib Resistance

**Figure 6:**
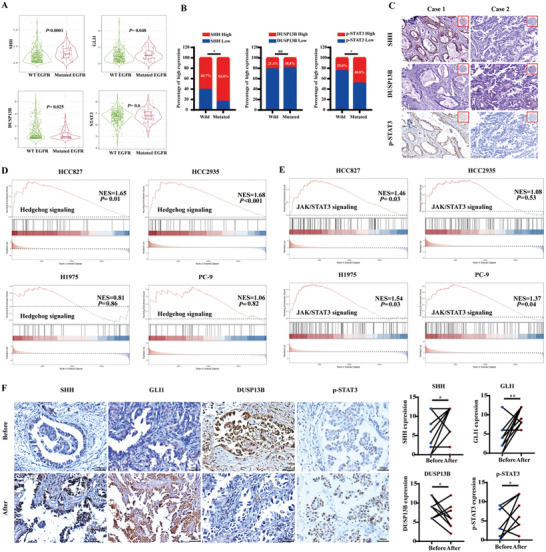
The expression of the SHH/DUSP13B/p‐STAT3 signaling axis. A) The correlation between SHH, GLI1, PTCH1, and DUSP13B with the mutation status of the EGFR gene. B) The relationship between SHH, DUSP13B, and p‐STAT3 proteins and the EGFR gene mutation status. C) The localization and expression of SHH, DUSP13B, and p‐STAT3 proteins in lung adenocarcinoma tissues. D) GEO database validated the involvement of Hedgehog pathway in osimertinib resistance. E) GEO database validated the involvement of JAK/STAT3 pathway in osimertinib resistance. F) The protein expression of SHH, GLI1, DUSP13B, and p‐STAT3 in lung cancer tissues before and after osimertinib resistance were examined with IHC staining. Data were presented as mean ± SD, and the *P* value was calculated using the wilcoxon signed‐rank test and chi‐squared test. *P < 0.05; **P < 0.01.

We first investigated the correlation between SHH, GLI1, DUSP13B, and STAT3, and the mutation status of the EGFR gene using the TIMER2.0 database (**Figure** **6**A). Surprisingly, the mRNA levels of SHH and GLI1 were markedly upregulated in lung adenocarcinoma tissues with EGFR mutations (*P* < 0.0001 and *P* = 0.048, respectively). Conversely, the mRNA level of DUSP13B was downregulated in lung adenocarcinoma tissues with EGFR mutations (*P* = 0.025). We then utilized the GEPIA2 online platform to examine the correlation among the mRNA levels of SHH, GLI1, DUSP13B, and STAT3 in lung adenocarcinoma tissues from the TCGA dataset. The results revealed significant positive correlations between SHH with GLI1, and STAT3 (*r* = 0.34, *P* < 0.0001; r = 0.16, *P* = 0.00 032) but a notable negative correlation with DUSP13B (*r* = −0.19, *P* < 0.0001). Additionally, a positive correlation between GLI1 and STAT3 (*r* = 0.15, *P* = 0.00 093) and a negative correlation between DUSP13B (*r* = −0.16, *P* = 0.00 054) were observed. Additionally, our findings revealed a pronounced negative association between the levels of STAT3 and DUSP13B (*r* = −0.11, *P* = 0.016; Figure S7, Supporting Information).

In addition, we conducted IHC staining on 108 lung adenocarcinoma tissues and assessed the association between the protein levels of SHH, DUSP13B, p‐STAT3, and EGFR gene mutation status. The results (Figure 6B) revealed that the expression rate of SHH protein exhibited a substantial increase in lung adenocarcinoma tissues with EGFR gene mutations (*P* = 0.012; 83.8%). Moreover, a higher expression rate of p‐STAT3 protein was observed in EGFR mutated‐type lung adenocarcinoma tissues (*P* = 0.029). Nevertheless, no significant correlation was observed between DUSP13B protein levels and the presence of EGFR mutations (*P* > 0.05). Subsequently, we analyzed the correlation between SHH, DUSP13B, and p‐STAT3 proteins. As shown in **Table** **2** and Figure 6C, we found a notable positive correlation between p‐STAT3 and SHH (*r* = 0.235, *P* = 0.014) but no significant correlation between p‐STAT3 and DUSP13B (*r* = 0.038, *P* = 0.700). Besides, we found a negative correlation between p‐STAT3 and DUSP3B (*r* = −0.234, *P* = 0.015).

**Table 2:** The association among expression of SHH, DUSP13B, and p‐STAT3 proteins in lung adenocarcinoma.

	SHH	DUSP13B	p‐STAT3
SHH			
Spearman's correlation coefficient	1	*r* = 0.038	*r* = 0.235
Sig. (2‐tailed)		*P* = 0.700	*P* = 0.014
DUSP13B			
Spearman's correlation coefficient	*r* = 0.038	1	*r* = −0.234
Sig. (2‐tailed)	*P* = 0.700		*P* = 0.015
p‐STAT3			
Spearman's correlation coefficient	*r* = 0.235	*r* = −0.234	1
Sig. (2‐tailed)	*P* = 0.014	*P* = 0.015	

Furthermore, we downloaded the osimertinib resistance dataset GSE193258 and conducted a GSEA enrichment analysis of the acquired data. This dataset contains transcriptome sequencing results before and after osimertinib resistance in four cell lines with EGFR mutations (PC9, HCC827, H1975, and HCC2935). GSEA enrichment analysis revealed an enrichment of the Hedgehog signaling pathway in osimertinib‐resistant HCC827 and HCC2935 cells (Figure 6D), as well as an enrichment of the JAK/STAT3 pathway in osimertinib‐resistant HCC827, H1975, and PC‐9 cells (Figure 6E).

To accurately determine the expression level of the SHH/DUSP13B/p‐STAT3 axis in osimertinib‐resistant lung cancer tissues, we collected 10 pairs of clinical lung cancer tissue specimens before and after osimertinib resistance and conducted IHC staining. As shown in Figure 6F, the SHH protein was predominantly expressed in the cell membrane and cytoplasm, with higher protein expression after osimertinib resistance than before. Both GLI1 and p‐STAT3 were primarily expressed in the nucleus and cytoplasm, with higher levels observed in postresistant tissues. Furthermore, DUSP13B protein was mainly expressed in the cytoplasm, with lower expression levels observed in postosimertinib‐resistant lung cancer tissues.

## 4 Discussion

EGFR is a well‐known driver gene in lung cancer, with a significant percentage of patients carrying mutations in this gene.^[^ ^30^, ^31^ ^]^ The third‐generation EGFR‐TKI osimertinib is an effective solution for overcoming resistance triggered by the T790M mutation, a common occurrence following treatment with first‐ and second‐generation TKIs. However, acquired resistance to osimertinib eventually develops, limiting its long‐term clinical utility.^[^ ^32^, ^33^ ^]^ Thus, there is a pressing need to investigate the mechanisms underlying osimertinib resistance, identify strategies to overcome it, and ultimately enhance the prognosis of patients with lung cancer.

Currently, therapies have been developed to tackle osimertinib resistance in combination with other drugs.^[^ ^34^, ^35^, ^36^ ^]^ We identified itraconazole as a significant inhibitor of osimertinib‐resistant cell proliferation in preliminary screening. Therefore, we investigated whether itraconazole can reverse osimertinib resistance and its specific molecular mechanisms. Initial assessment of the Combination Index indicated a potent synergistic effect of itraconazole combined with osimertinib, which was supported by subsequent in vitro experiments demonstrating synergistic effects on various malignant phenotypes of osimertinib‐resistant cells. Further validation in vivo confirmed the therapeutic efficacy of this combination in inhibiting the growth of osimertinib‐resistant tumors without significant toxic side effects. Ki‐67 staining (a proliferation marker^[^ ^37^ ^]^) revealed a significant reduction in tumor cell proliferation, reinforcing the observed antiproliferative effects.

To clarify the molecular mechanisms of the synergistic effects, we conducted RNA‐seq and revealed the modulation of the Hh and JAK/STAT signaling pathways. Subsequent western blot experiments confirmed the reduced activity of these pathways upon combination treatment. SHH is a crucial ligand of the Hh pathway, playing a pivotal role in cell differentiation, and proliferation.^[^ ^38^ ^]^ In lung cancer tissues, the expression level of the SHH protein was significantly and positively correlated with p‐STAT3.^[^ ^39^ ^]^ We then decided to further explore whether there is a specific regulatory relationship between them, thereby linking the Hh and STAT3 pathways. The results showed that upregulation of SHH led to the activation of p‐STAT3 and increased the expression of its target protein c‐Myc. For the first time, our investigation reveals the modulatory impact of SHH on the STAT3 pathway and provides initial insights into the existence of the SHH/p‐STAT3 signaling axis.

As SHH has not been reported to possess kinase or phosphatase activity, we speculated that other kinases or phosphatases might be involved in regulating the SHH/p‐STAT3 signaling axis in response to itraconazole combined with osimertinib. Initially, we evaluated the expression levels of JAKs proteins, known for their association with STAT3, but found that the combination of itraconazole with osimertinib did not mediate its effects through the JAKs kinases. DUSPs play a pivotal role in cellular physiology by dephosphorylating phosphoserine, phosphothreonine, and phosphotyrosine residues in proteins.^[^ ^28^ ^]^ We observed a substantial increase in several DUSPs after itraconazole treatment. We then conducted correlation analysis, qPCR, and western blotting, focusing on DUSP13B. The data demonstrated a pronounced upregulation of both mRNA and protein of DUSP13B following itraconazole combined with osimertinib treatment. To ascertain whether DUSP13B could dephosphorylate STAT3, we conducted in vivo phosphorylation experiments and illustrated dephosphorylation by DUSP13B. Molecular docking has been widely utilized to predict interactions between proteins.^[^ ^40^ ^]^ In this study, we conducted molecular docking of STAT3 and DUSP13B using the online website ZDOCK^[^ ^41^ ^]^ to predict their interactions. Employing molecular docking and subsequent co‐IP and immunofluorescence experiments, we confirmed the interaction between DUSP13B and STAT3.

DUSPs play an important role in tumorigenesis and drug resistance.^[^ ^42^, ^43^, ^44^, ^45^, ^46^ ^]^ Here, we investigated the involvement of DUSP13B in osimertinib resistance. Our findings revealed a significant reduction in DUSP13B expression after osimertinib resistance, and overexpression of DUSP13B reversed osimertinib resistance and enhanced the cell sensitivity to osimertinib. Moreover, we observed that combined treatment with itraconazole and osimertinib facilitated SHH protein degradation, leading to reduced SHH expression. Overexpression of SHH partially counteracted the synergistic effects of the drug combination, both in vitro and in regulating the SHH/DUSP13B/p‐STAT3 signaling axis. These findings collectively shed light on the molecular mechanisms underlying the synergistic effects of itraconazole and osimertinib, and provide compelling evidence for the therapeutic strategy of combining itraconazole with osimertinib to overcome osimertinib resistance in NSCLC.

Autophagy is a highly conserved degradation process that contributes significantly to the maintenance of cellular homeostasis. It plays a dual role in the cancer development, metastasis, and drug resistance.^[^ ^22^, ^47^ ^]^ Therefore, investigating whether autophagy is induced during drug treatment and understanding its role are crucial for enhancing antitumor efficacy. Through analysis of the RNA‐seq results, we observed significant enrichment of the autophagy pathway in the itraconazole‐treated group. LC3 is involved in the development of autophagosomes, and the level of autophagy is reflected by the ratio of LC3II/LC3I.^[^ ^48^, ^49^, ^50^ ^]^ P62 protein, encoded by the *SQSTM1* gene, is known to localize to autophagosomes and lysosomes, playing a crucial role as an autophagy adaptor protein that can also reflect autophagy activity.^[^ ^51^ ^]^ According to our examination, we found that granular LC3 was markedly increased in the combination therapy group, suggesting an increase in autophagosomes. Additionally, the ratio of LC3II/LC3I and P62 were also significantly increased. It is commonly assumed that increased P62 expression indicates the inhibition of autophagy. However, under toxic stimulation and oxidative stress, the expression level of P62 protein may align with autophagy activation. This is because P62 is also a stress protein that is significantly elevated under stressful conditions.^[^ ^51^, ^52^ ^]^ Therefore, the activity of cellular autophagy cannot be determined based solely on a single experimental result. Observing the cellular ultrastructure through TEM is crucial for directly visualizing autophagosomes. Our observations revealed a notable rise in the count of autophagosomes in cells treated with the combination of itraconazole and osimertinib. Autophagy is a dynamic process that involves the formation of autophagosomes, their fusion with lysosomes, and the degradation of autolysosomes.^[^ ^48^, ^53^ ^]^ By monitoring intracellular fluorescence changes following infection with mRFP‐GFP‐LC3, we can observe the cellular autophagic flux more vividly and clearly, which serves as a reliable indicator to accurately assess autophagic activity.^[^ ^54^ ^]^ Lysosomes are an acidic environment. When the autophagosome fuses with the lysosome, the green fluorescence (GFP) on the autophagosome is quenched, resulting in only red fluorescence (mRFP), indicating unobstructed autophagic flow. If the acidic environment within the lysosome is disrupted or the autophagosome fails to fuse with the lysosome, the GFP green fluorescence will not be quenched. This leads to a large amount of yellow fluorescence within the cell, indicating blocked autophagic flow.^[^ ^55^ ^]^ Assessment of autophagy markers revealed increased autophagic activity following the combination therapy. The inhibitor chloroquine further improved the synergistic effects of itraconazole and osimertinib, indicating that induced autophagy serves as a protective mechanism, and inhibiting it could enhance the antitumor efficacy of the combination.

EGFR is a transmembrane glycoprotein receptor belonging to the tyrosine kinase receptor family with a molecular weight of 170 kDa. EGFR gene mutations can lead to aberrant activation of a cascade of downstream pathways, such as JAK/STAT, PI3K/Akt, and MAPK, leading to tumor development.^[^ ^56^ ^]^ We investigated the correlation between key molecules, including SHH, GLI1, DUSP13B, and STAT3, and the mutation status of EGFR using the TIMER2.0 online website. Our findings revealed that the mRNA expression levels of SHH and GLI1 were markedly increased in the EGFR mutation group, whereas DUSP13B was greatly decreased. These results suggest that EGFR mutations may trigger the activation of the Hedgehog signaling pathway, potentially posing a latent risk for EGFR‐TKI resistance. To further explore the expression levels and correlation of SHH/DUSP13B/p‐STAT3 in lung adenocarcinoma tissues, we performed IHC staining and found that the high expression rate of SHH protein in EGFR‐mutant lung adenocarcinoma tissues was significantly higher than that in EGFR wild‐type tissues, which was consistent with the mRNA expression pattern. These results are consistent with the findings reported by Kim et al., who observed higher expression of SHH in lung adenocarcinoma patients with EGFR‐mutant, which was related to a poor prognosis.^[^ ^57^ ^]^ Furthermore, the high expression rate of p‐STAT3 protein was significantly increased in EGFR‐mutant lung adenocarcinoma tissues. However, DUSP13B protein expression was not related to EGFR mutation status, which may be attributed to the relatively small sample size. We also analyzed the association between SHH, DUSP13B, and p‐STAT3 proteins in lung adenocarcinoma tissues and found that p‐STAT3 was significantly positively correlated with SHH but negatively correlated with DUSP13B. This further suggests that DUSP13B may have a negative regulatory relationship with p‐STAT3, and further verification of this finding is needed in other types of cancer tissues in future studies.

**Figure 7:**
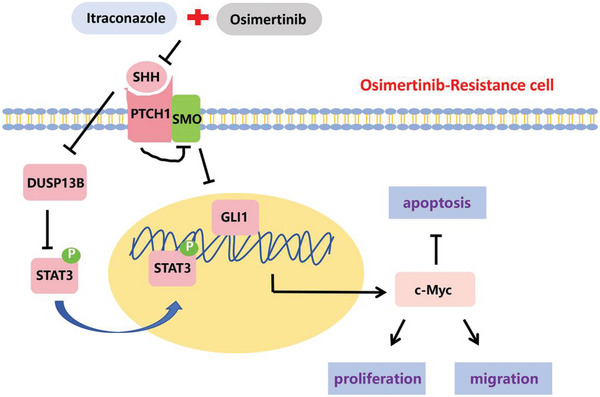
Schematic diagram of molecular mechanism of synergistic effects of itraconazole in combination with osimertinib.

Furthermore, we verified that the Hh and JAK/STAT3 signaling pathways were implicated in osimertinib resistance using the GEO dataset. However, it is worth noting that variations may exist across different cell types. In addition, we observed a considerable increase in SHH protein expression in lung cancer tissues after osimertinib resistance compared with that in preresistant tissues. Given that the SHH protein is a secreted molecule detectable in serum, it could serve as a valuable marker for noninvasively predicting osimertinib resistance. Moreover, our study highlights the clinical relevance of these findings, demonstrating the increased expression of SHH, p‐STAT3, and GLI1 proteins in postosimertinib‐resistant lung cancer tissues, along with decreased DUSP13B expression. These proteins may serve as valuable molecular markers for predicting osimertinib resistance. And the working model was shown in **Figure** **7**.

Overall, our comprehensive investigations significantly contribute to the understanding of the molecular mechanisms underlying osimertinib resistance and shed light on potential therapeutic strategies for overcoming this resistance in NSCLC.

## Conflict of Interest

The authors declare no conflict of interest.

## Author Contributions

H.Z.: Resources, software, formal analysis, writing‐original draft, writing‐review and editing. Y.T.: Data curation, and validation. H.Z.: Funding acquisition, and methodology. J.L.: Software, and funding acquisition. H.Z.: Investigation, and methodology. Y.Z.: Software. Y.Z.: Formal analysis. Q.W.: Resources. J.M.: Supervision, investigation. S.F.: Conceptualization, data curation, supervision, funding acquisition, visualization, writing‐review, and editing.

## Supporting information



Supporting Information

## Data Availability

The data that support the findings of this study are available from the corresponding author upon reasonable request.
