# Supplementary material for: Itraconazole Reversing Acquired Resistance to Osimertinib in NSCLC by Inhibiting the SHH/DUSP13B/p‐STAT3 Axis
Source: Adv Sci (Weinh). 2024 Dec 25;12(7):2409416. doi: 10.1002/advs.202409416 (PMC11831513; doi:10.1002/advs.202409416)
Supplement: Supplementary file 1 — Supporting Information [file ADVS-12-2409416-s001.docx]

**Table S1 Summary of Antibody Information.**

| Antibody | Cat No. | Dilution |
| --- | --- | --- |
| SHH | ZEN-BIOSCIENCE(R25772) | 1:1000 |
| c-PARP | CST(#9541) | 1:500 |
| Mcl-1 | CST(#4572) | 1:1000 |
| Bax | CST (#5023) | 1:1000 |
| Bcl-2 | ZEN-BIOSCIENCE (R23309) | 1:1000 |
| Bcl-xL | CST (#2764) | 1:1000 |
| N-cadherin | Proteintech (22018-1-AP) | 1:2000 |
| GAPDH | Proteintech (60004-1-Ig) | 1:5000 |
| MMP-9 | Abclonal (A0289) | 1:1000 |
| MMP-2 | Abclonal (A6247) | 1:1000 |
| p-STAT3 | ZEN-BIOSCIENCE (381552) | 1:1000 |
| c-Myc | ZEN-BIOSCIENCE (R22809) | 1:1000 |
| GLI1 | Proteintech (66905-1-Ig) | 1:5000 |
| DUSP13B | Proteintech (10909-1-AP) | 1:1000 |
| STAT3 | Proteintech (60199-1-Ig) | 1:1000 |
| JAK1 | Proteintech (66466-1-Ig) | 1:1000 |
| Flag | Proteintech (66008-4-Ig) | 1:1000 |
| β-actin | Proteintech (81115-1-RR) | 1:1000 |
| PTCH1 | Elabscience (E-AB-10571) | 1:1000 |
| JAK2 | CST (#3230) | 1:1000 |
| TYK2 | Abclonal (A2128) | 1:1000 |
| P62 | ZEN-BIOSCIENCE (R27312) | 1:1000 |
| LC3 | Proteintech (14600-1-AP) | 1:1000 |
| α-tublin | Proteintech (11224-1-AP) | 1:1000 |

**Table S2 Summary of primer sequences.**

| Gene | primer sequence (5’-3’) |
| --- | --- |
| GAPDH | F: CAGGAGGCATTGCTGATGAT |
|  | R: GAAGGCTGGGGCTCATTT |
| DUSP13B | F: GCTGCCACACTGAACCATATC |
|  | R: GGACATTCCACGGTAGAATTTGG |

**
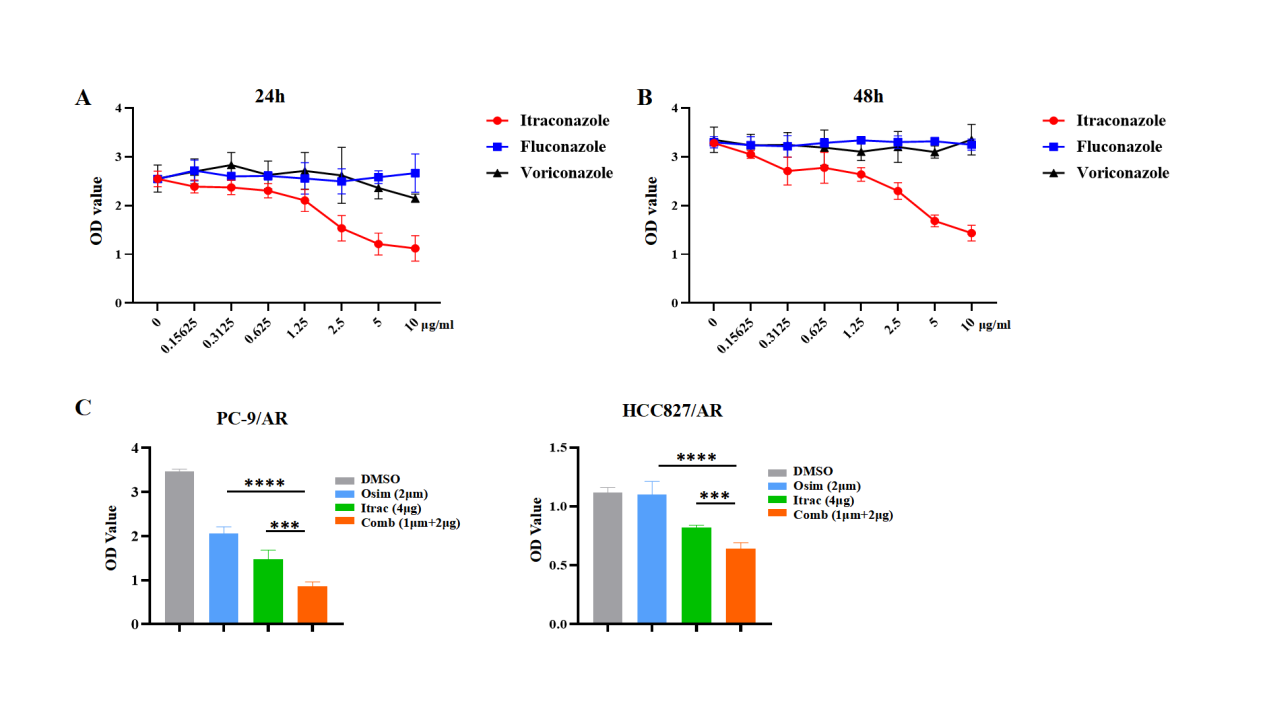
**

**Figure S1. Screening for antifungal drugs that could inhibit the proliferation of osimertinib-resistant cells.** (A) The effect of different antifungal drugs on the proliferation ability of PC-9/AR cells 24 hours after treatment. (B) The effect of different antifungal drugs on the proliferation ability of PC-9/AR cells 48 hours after treatment. (C) The effect of combining itraconazole and osimertinib compared to double-dose single drug treatment on the proliferation of PC-9/AR and HCC827/AR cells. Data were presented as mean±SD (n=3), and the *P* value was calculated using unpaired student's t-tests. ***, *P<*0.001; ****, *P*<0.0001.

**
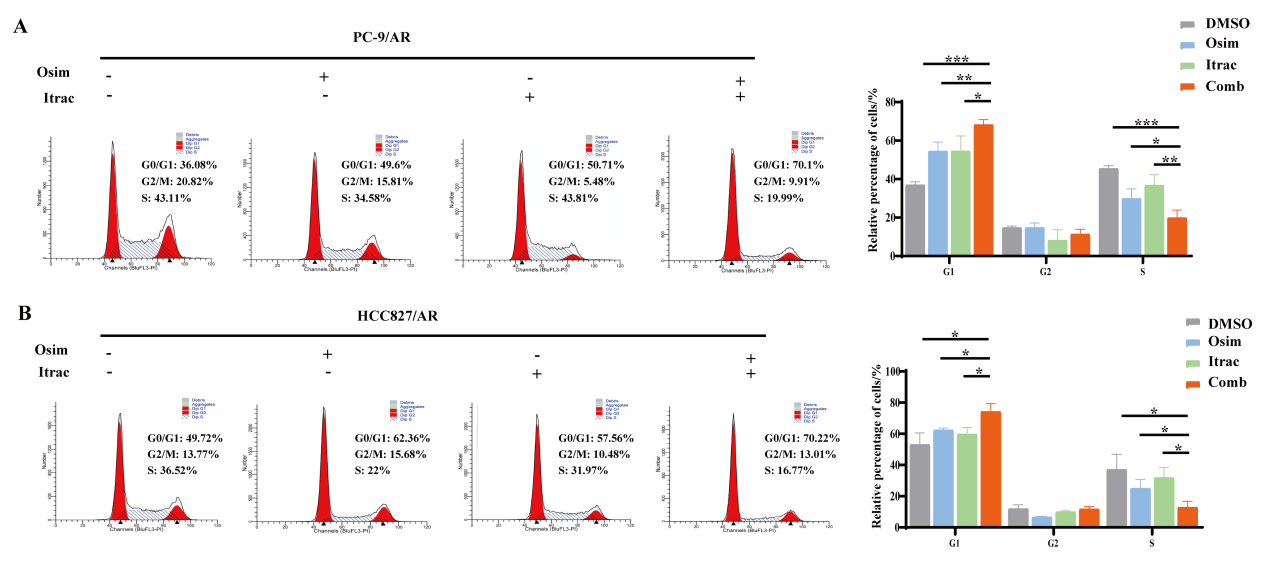
**

**Figure S2. Effect of itraconazole combined with osimertinib on the cell cycle detected by flow cytometry.** (A) Itraconazole combined with osimertinib synergistically induced G0/G1 phase arrest and S phase reduction in PC-9/AR cells. (B) Itraconazole combined with osimertinib synergistically induced G0/G1 phase arrest and S phase reduction in HCC827/AR cells. Osim: osimertinib; Itrac: itraconazole; Comb: the combination of osimertinib and itraconazole. Data were presented as mean±SD (n=3), and the *P* value was calculated using unpaired student's t-tests. *, *P*<0.05; **, *P*<0.01; ***, *P*<0.001.

**
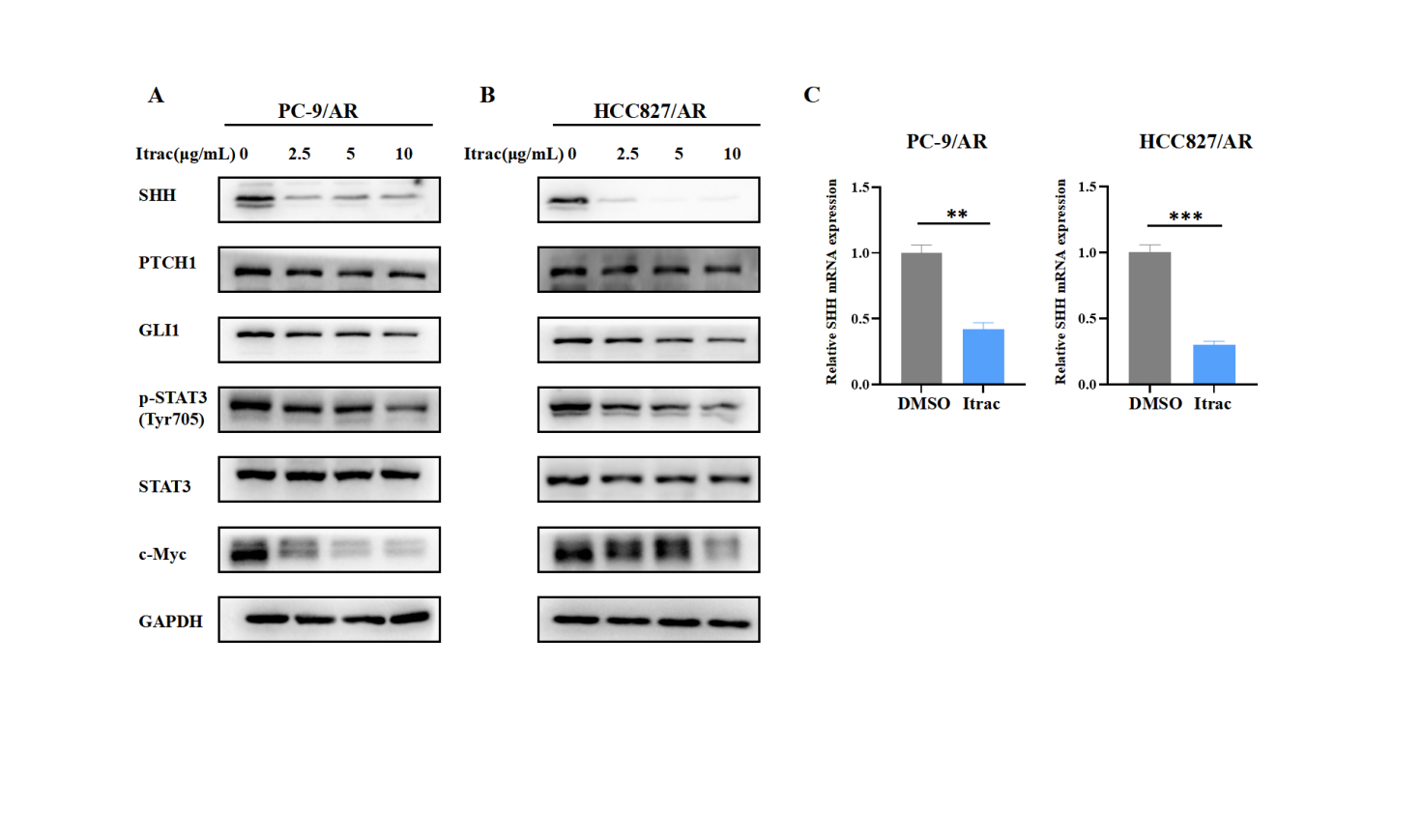
**

**Figure S3.** **Itraconazole modulated Hedgehog and STAT3 signaling pathway.** (A) Itraconazole modulated the key proteins of Hedgehog and STAT3 signaling pathway in PC-9/AR cells. (B) Itraconazole modulated the key proteins of Hedgehog and STAT3 signaling pathway in HCC827/AR cells. (C) The mRNA expression levels of SHH after treatment with itraconazole. Data were presented as mean±SD (n=3), and the *P* value was calculated using unpaired student's t-tests. **, *P*<0.01; ***, *P*<0.001.

**
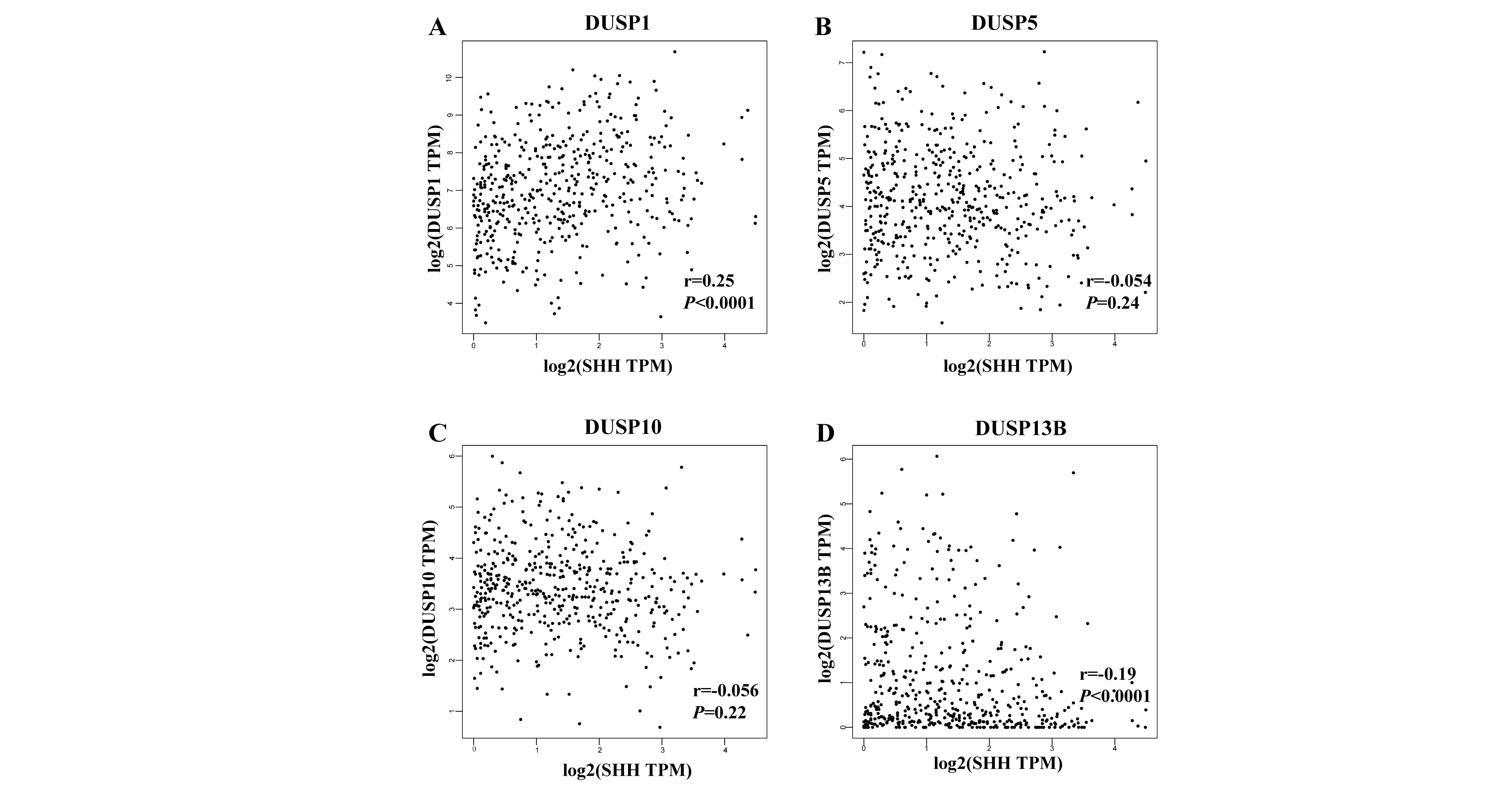
**

**Figure S4. The correlation between DUSPs and SHH.** (A) DUSP1 was significantly positively correlated with SHH (r=0.25, *P*<0.0001). (B) There was no correlation found between DUSP5 and SHH (r=-0.054，*P*=0.24). (C) There was no correlation found between DUSP10 and SHH (r=-0.056, *P*=0.22). (D) DUSP13B was significantly negatively correlated with SHH (r=-0.19, *P*<0.0001). *P* value was calculated using spearman's coefficient.

**
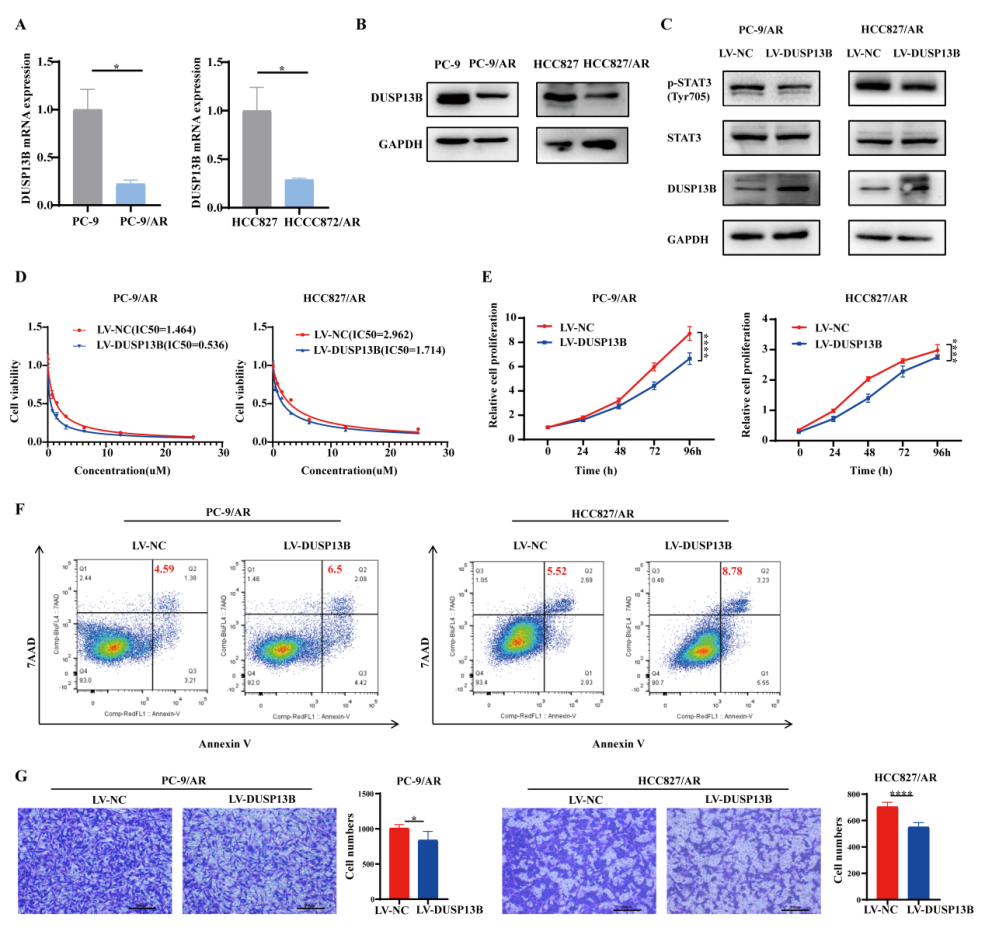
**

**Figure S5. Overexpression of DUSP13B reversed osimertinib resistance in NSCLC.** (A and B) The expression of DUSP13B was detected by qPCR and western blotting. (C) The successful construction of DUSP13B overexpression was confirmed by western blotting. (D) Overexpression of DUSP13B reduced the IC50 in PC-9/AR and HCC827/AR cells. (E) Overexpression of DUSP13B inhibited the proliferation of PC-9/AR and HCC827/AR cells. (F) Overexpression of DUSP13B promoted the apoptosis of PC-9/AR and HCC827/AR cells. (G) Overexpression of DUSP13B inhibited the migration of PC-9/AR and HCC827/AR cells. LV-NC: the control group; LV-DUSP13B: stable overexpression of the DUSP13B group. Data were presented as mean±SD (n=3), and the *P* value was calculated using unpaired student's t-tests and two-way ANOVA test. *, *P*<0.05; ****, *P*<0.0001.

**
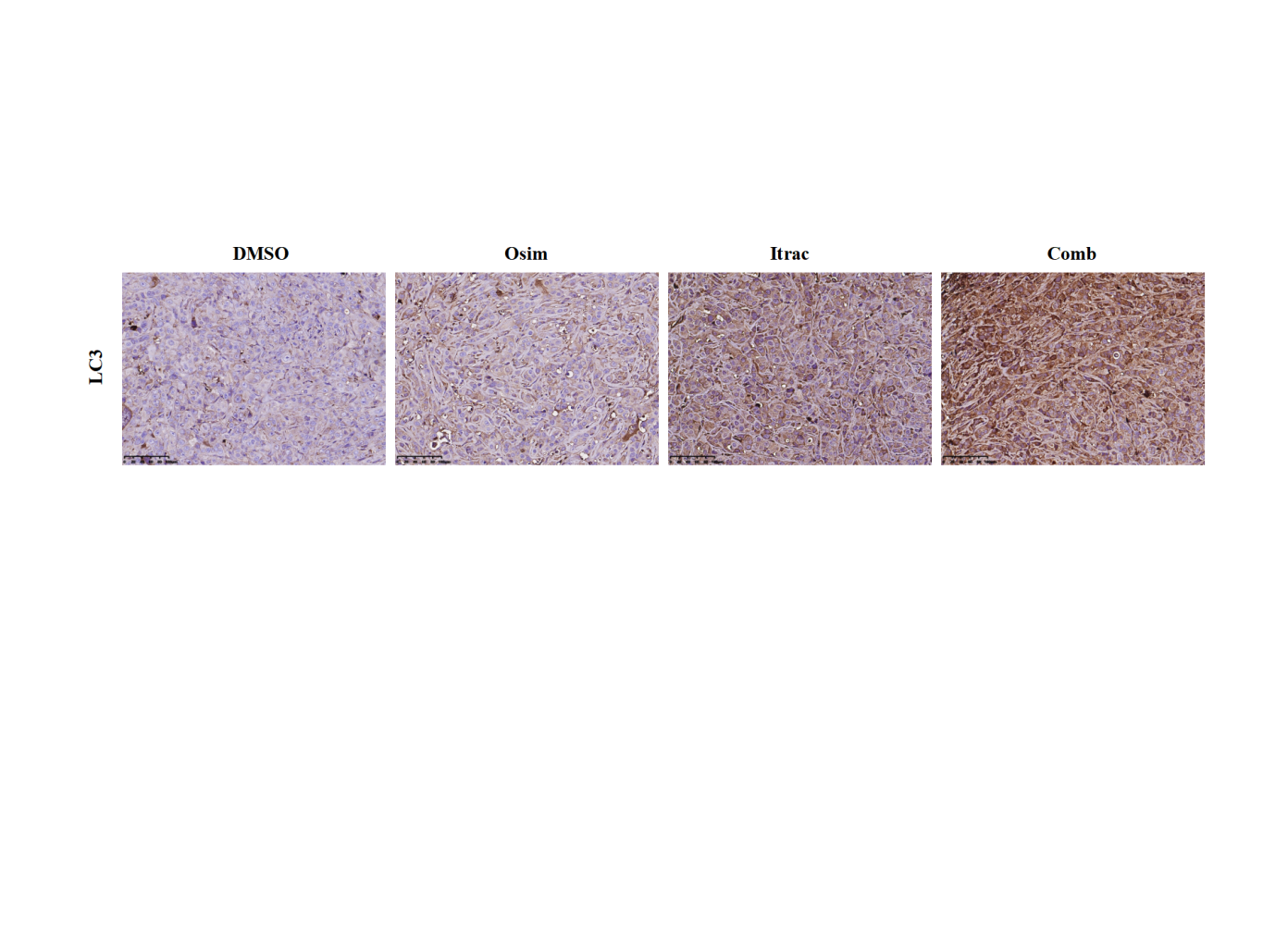
**

**Figure S6. Representative images of IHC staining of autophagy protein.** LC3 protein expression was highest in the combined treatment group. Osim: osimertinib; Itrac: itraconazole; Comb: the combination of osimertinib and itraconazole.

**
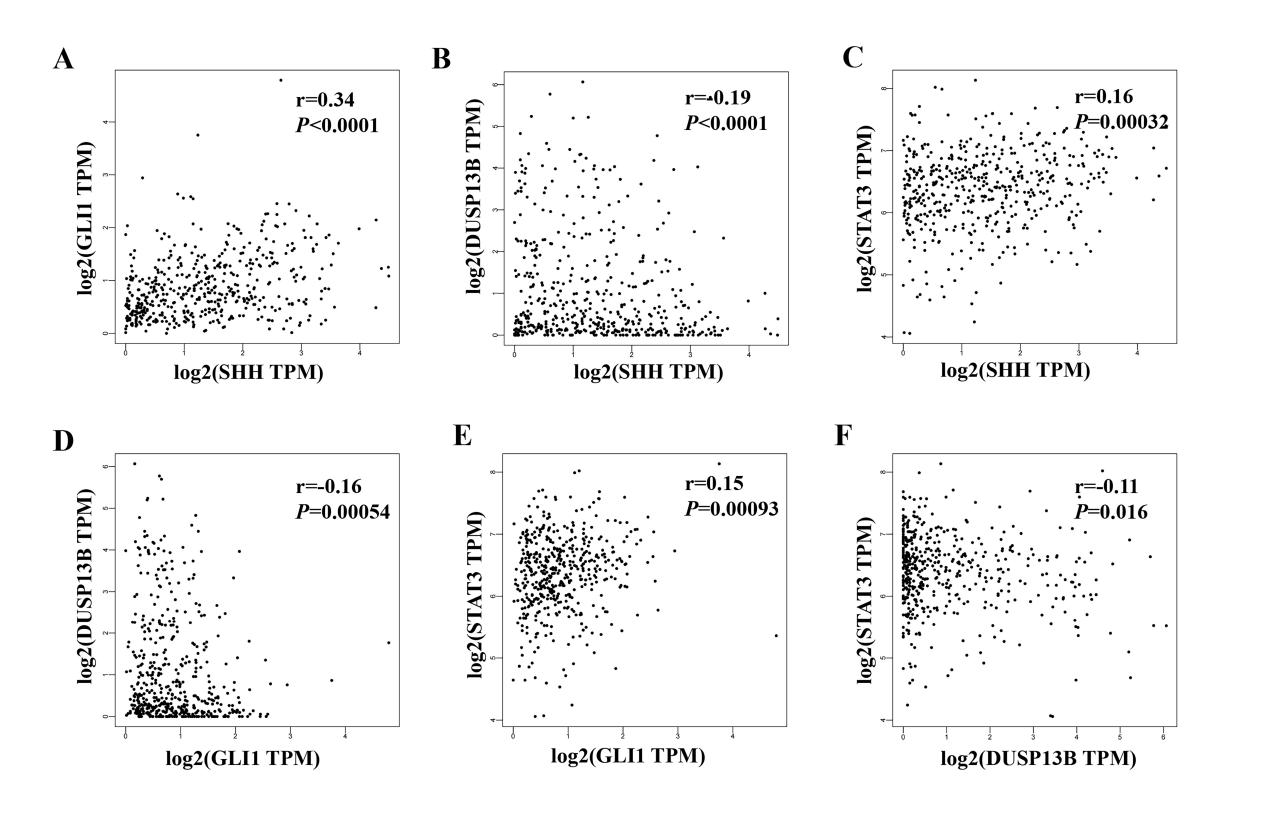
**

**Figure S7. The correlation among the mRNA expression levels of SHH, GLI1, DUSP13B, and STAT3.** (A) The correlation between SHH and GLI1 (r=0.34, *P*<0.0001). (B) The correlation between SHH and DUSP13B (r=-0.19, *P*<0.0001). (C) The correlation between SHH and STAT3 (r=0.16, *P=*0.00032). (D) The correlation between GLI1 and DUSP13B (r=-0.16, *P=*0.00054). (E) The correlation between GLI1 and STAT3 (r=0.15, *P=*0.00093). (F) The correlation between STAT3 and DUSP13B (r=-0.11, *P=*0.016). *P* value was calculated using spearman's coefficient.
